# Examining Delineated Competencies within Blended Hospital/Health System Pharmacy and General Medicine Advanced Pharmacy Practice Experiences

**DOI:** 10.3390/pharmacy12040124

**Published:** 2024-08-13

**Authors:** Jennifer L. Prisco, Yulia A. Murray, Tewodros Eguale, Jennifer D. Goldman

**Affiliations:** School of Pharmacy, Massachusetts College of Pharmacy and Health Sciences, Boston, MA 02115, USAjennifer.goldman@mcphs.edu (J.D.G.)

**Keywords:** institutional, general medicine, blended rotation, longitudinal, experiential, advanced pharmacy practice experience

## Abstract

In the United States, Doctor of Pharmacy (PharmD) programs are required to provide advanced pharmacy practice experiences (APPEs) in the core inpatient rotation areas of hospital/health system pharmacy and inpatient general medicine patient care. Colleges and Schools of Pharmacy (C/SOPs) nationwide are increasingly utilizing blended or longitudinal APPE models to offer experiential opportunities; however, there is a gap in the literature to support programs with delineating rotation-specific competencies when integrating two or more rotations together. Utilizing a survey instrument, PharmD students at two C/SOPs reported their onsite inpatient rotation sub-competency activities achieved within the four competency areas of Hospital/Health Pharmacy Systems, Medication Safety and Quality, Clinical Applications, and Professional Practice, which are listed in Appendix C of the 2016 Accreditation Council for Pharmacy Education Standards Guidance Document. Unpaired two-sample *t*-tests were performed to compare proportions of sub-competency activity occurrence in the two rotation settings. In total, 168 students reported inpatient activities related to the four competency areas, with 95–100% reporting their involvement in one or more sub-competency opportunities within each area. Of the 26 sub-competencies compared, 73% significantly facilitated the development of competency to a greater extent for one APPE inpatient rotation type over the other (*p* < 0.05). The findings can be utilized by C/SOPs to support the delineation of rotation-specific competencies when blending inpatient experiential opportunities.

## 1. Introduction

In the United States (U.S.), learners in a Doctor of Pharmacy (PharmD) program are required to complete, at minimum, 1740 h of experiential learning as part of the curriculum [1]. The Accreditation Council for Pharmacy Education (ACPE) is the accreditor for U.S. PharmD programs and requires at least 300 h of introductory pharmacy practice experiences (IPPEs) and 1440 h of advanced pharmacy practice experiences (APPEs). Within APPEs, a minimum of 4 weeks (160 h) must be spent in each of the following direct patient care settings: (1) ambulatory patient care, (2) community pharmacy, (3) hospital/health system pharmacy (HS), and (4) inpatient general medicine patient care (GM). The first two practice settings must be in the outpatient setting, while the latter two must be in the inpatient setting. While ACPE is clear on the four required patient care experiences and designates them to occur in either the inpatient or the outpatient setting, the burden is on individual Colleges/Schools of Pharmacy (C/SOPs) running the PharmD program to establish both general and experience-specific learning outcomes for each required APPE. Schools must identify the specific competencies to be achieved and the setting needed to meet outcomes. ACPE also put out draft revised standards in January 2024 that continue to have these four core rotations required [2]. Of note, the GM rotation has been renamed to adult inpatient care within the draft standards. In both the current and draft ACPE standards, specific definitions and criteria are not provided for programs to utilize when defining HS and GM APPE rotations.

Within U.S. PharmD standards, ACPE acknowledges the potential blurring of pharmacy practice settings where competency attainment can be achieved in more than one setting type [1]. The literature shows blended or longitudinal APPE models are a growing opportunity utilized by C/SOPs [3,4,5,6,7]. With blended models, two or more required APPE types are intermingled [1]. As an example, students may participate for 20 h a week in one functional area of the health system (e.g., morning rounding with a healthcare team) and then participate for 20 h a week in an alternative functional area (e.g., compounding and hospital stewardship initiatives). Blended models could also include dividing experiential time within a week or rotation block to serve the outpatient population in an ambulatory care clinic while also spending time supporting the inpatient population by monitoring lab parameters, as another example. Typically, if offering blended APPEs, the experience will be no less than 8 weeks total to ensure the APPE requirements of two rotation types are being addressed. Blended models do not necessarily have to be within the same health system. With longitudinal models, two or more required APPE types are occurring within the same health system [1]. Longitudinal models can integrate the blending of different rotation types, or they can be standalone rotations completed within different areas of the health system in back-to-back rotation blocks. Longitudinal models are useful to health systems and to experiential departments to help minimize onboarding paperwork for students. It is less burdensome to onboard a single student to participate at a site for 12 weeks than it is to onboard two students who will be there for 6 weeks each, for example. When using either of these models in PharmD experiential education, schools must still be able to document “how its APPE program is balanced between the four required practice areas and how all program outcomes, student performance competencies, and ACPE standards are met” [1].

During the literature search performed to evaluate (1) longitudinal APPE models, (2) blended APPE models, and (3) current competency delineation in blended and/or longitudinal APPE models, no results were produced for competency delineation. There is literature discussing (1) the establishment of longitudinal APPE models where students complete required hospital practice and acute patient care rotations within the same health system and (2) what steps are required to set up longitudinal experiences [3,4,5,6,7]. However, there is no identified discussion of how competencies are attained and delineated during each experience. In 2019, a U.S.-based Task Force within the American Association of Colleges of Pharmacy (AACP) Experiential Education Section (EES) published a consensus-building, peer-reviewed set of APPE essential elements that could be used to bring consistency and rotation competency delineation for rotation experiences [8]. This Task Force was able to build consensus for GM based on what students could and should be able to do while on the rotation; however, agreement was not attained regarding essential elements for HS based on varying opinions related to “whether the experience should primarily be in direct patient care, distribution/operations, or management”.

The purpose of this paper is to outline how two C/SOPs utilized the work completed by the American Society of Health-System Pharmacists (ASHP) and ACPE joint Task Force, *Entry-level Competencies Needed for Pharmacy Practice in Hospitals and Health-Systems*, to characterize rotation-specific competencies for inpatient-required APPEs [9]. By identifying competencies and sub-competencies that are more applicable to one inpatient-required rotation over the other, this information can be utilized when implementing longitudinal or blended models to delineate rotation-specific competencies, tasks, and outcomes. This information can also be used by programs during preceptor and site development to assist preceptors with building learner opportunities to become entry-level, practice-ready pharmacists. Notably, work completed by the ASHP–ACPE Task Force was adopted, verbatim, by the ACPE within its Appendix C of the 2016 ACPE Standards Guidance Document for U.S. C/SOPs to utilize as a resource, which is why authors have utilized this source to delineate competencies [10]. This guidance document has four competency areas within the categories of Hospital/Health Pharmacy Systems, Medication Safety and Quality, Clinical Applications, and Professional Practice and twenty-six sub-competencies that align to one of four overarching competencies.

To provide the context of what hospitals and health systems are like in the U.S., inpatient hospital settings often have a centralized pharmacy where medication orders and fulfillment are completed. This may include common pharmacist roles in order verification, triaging medication deliveries to the patient floors, controlled substance dispensing, compounding sterile and non-sterile medication products, formulary decision making involvement, stewardship programs, inventory management, and direct oversight of pharmacy support staff. There are also commonly decentralized pharmacist roles within larger inpatient hospitals where pharmacists round with a team of healthcare providers, such as doctors and nurses, to make recommendations on medication-related interventions, verify medication orders, ensure adherence to hospital medication guidelines, and contribute to direct patient care. For the two participating programs in this study, the decentralized pharmacist’s role is more suitable for GM experiences and the centralized pharmacy is more compatible with the HS experience.

## 2. Materials and Methods

Data were collected from the Class of 2020 PharmD students who were in the last year of their program at two separately accredited U.S. C/SOPs. Both programs were at a single, multi-campus university with a centralized pharmacy experiential education office and a centralized Institutional Review Board to approve thisstudy. One accredited program was a year-round accelerated professional PharmD curriculum with fall, spring, and summer semesters that allows a student to graduate in under 3 years, and the second program was a traditional U.S. 4-year professional PharmD program with fall and spring didactic semesters. These students had only completed one of the two required inpatient rotations at the time of reporting, either HS or GM. Each APPE offered at these C/SOPs was 6 weeks (240 h) in length. In both programs, APPEs are completed after introductory rotations and didactic courses have been successfully completed. No respondents were participating in a blended or longitudinal inpatient APPE model.

Using a Qualtrics^®^ survey, learners reported onsite inpatient sub-competency attainment within Appendix C of the 2016 ACPE Standards Guidance Document. Qualtrics^®^ version March 2020 is a cloud-based software platform that users can use to build and distribute survey instruments, and it is headquartered in Provo, Utah, USA. The survey administered included the sub-competencies listed within Appendix C of this ACPE document verbatim [10]. Students indicated which core inpatient rotation they completed, and this survey was administered at the beginning of the student’s second inpatient APPE. Students had to indicate which projects, consults, tasks, or other activities they completed in each of the 26 sub-competency categories, and they selected all sub-competencies that applied to their prior inpatient APPE experience. Responses from participants used in this study were either yes (achieved the sub-competency) or no (did not achieve the sub-competency). The developed survey allowed for calculating proportions for each sub-competency using the number of students that reported sub-competency attainment divided by the overall number of students per rotation type. Preceptors attested to the students’ reporting claims, and statistical analysis was performed to identify significant factors related to the inpatient rotation types.

The null hypothesis was that there is no difference in activity occurrence between APPE hospital/health system pharmacy and inpatient general medicine patient care settings. Unpaired two-sample *t*-tests were performed using SAS software version 9.4 to compare proportions of activity occurrence of the two core inpatient rotation settings, and *p*-values less than 0.05 were considered statistically significant. Furthermore, a threshold of >60%, taken together, was utilized as a cutoff when analyzing results to examine whether a particular sub-competency is both offered and attainable for achievement by learners throughout an inpatient experience.

This study was approved by the Institutional Review Board at Massachusetts College of Pharmacy and Health Sciences.

## 3. Results

In total, 168 APPE inpatient rotation reports were collected, with 51% (n = 86) being HS and 49% (n = 82) being GM. Of the 86 HS reports collected, 59% of participants (n = 51) were matriculating in the accelerated PharmD program. For the 82 GM participants, 51% (n = 42) were enrolled in the accelerated PharmD pathway. Of the 26 sub-competencies compared, 73% (n = 19) significantly facilitated the development of competency to a greater extent for one APPE inpatient rotation type over the other (*p* < 0.05). All sub-competencies are achievable at a >60% threshold on HS rotations, and 81% are achievable on GM rotations at this threshold.

### 3.1. Hospital/Health Pharmacy Systems

The Hospital/Health Pharmacy Systems competency includes eight sub-competencies. HS was favored in 100% of sub-competencies, with 88% being statistically significant (*p* < 0.05) when compared to GM. Of the eight sub-competencies, 63% were able to be achieved at a rate of 60% or greater for both APPE types. See Table 1.

### 3.2. Medication Safety and Quality

To evaluate the competency area of Medication Safety and Quality, five sub-competencies were appraised, with the majority (80%) occurring more frequently on HS. Three of the four sub-competencies that favored HS were statistically significant. Most sub-competencies (60%) can be achieved in both APPE inpatient settings at a frequency greater than 60%. See Table 2.

### 3.3. Clinical Applications

Sub-competencies related to Clinical Applications statistically favor their occurrence while on GM rotations (*p* < 0.05), with the exception of *contributing to the establishment of medication-use policies, criteria, and maintenance of the formulary within a Pharmacy and Therapeutics Committee using evidence-based approaches,* which is significantly more applicable to HS (*p* = 0.0189). Of the two sub-competencies statistically favoring occurrence in GM, they were still reportedly achievable 77–79% of the time while on HS. See Table 3.

### 3.4. Professional Practice

Upon evaluating the area of Professional Practice, there was no statistical difference in 50% of the sub-competencies, while 50% of sub-competencies favored achievement within GM (*p* < 0.05). Although statistical significance was determined for some sub-competencies, all 10 sub-competencies can be achieved in both rotation types at a rate above 60%, and the majority (90%) occur at a higher frequency while on GM. See Table 4.

Of the students reporting inpatient activities related to the four competency areas, 95–100% reported achieving one or more sub-competency in each of the four areas. Hospital/Health Pharmacy Systems sub-competencies had the most learners selecting “none of the above” for achievement at a range of 3–5%.

## 4. Discussion

While most sub-competency areas occur significantly more in one rotation type compared to the other, many can be achieved in both inpatient rotation types regardless of significance. This finding further supports the use of blended inpatient models, knowing competency attainment often occurs during both rotation types. Because U.S. accreditors do not set competency attainment thresholds, programs can select a threshold to implement in their experiential quality assurance process to monitor whether or not students are able to both (1) obtain the particular experience (i.e., it is offered at the site) and (2) develop competency in the area (i.e., students are given the opportunity to have hands-on practice to develop the competency). Not all rotation settings may offer the same opportunities, so it is imperative to have quality measures pre-designed and to monitor whether programs are meeting their goals. If goals are not being met, programs can initiate preceptor and site development to discuss learning opportunity needs.

Implementing a 60% threshold was used as a guide within the centralized experiential office to indicate that most of the rotation sites used by the two programs could both offer and allow sub-competency achievement to students; more than 60% of participating students on the rotation type had both the opportunity to (1) participate in the sub-competency (i.e., it is made available at the practice site) and (2) develop competency (i.e., students are integrated through tasks, assignments, projects, or other options to build competency). This threshold was used by the participating programs as part of a preceptor development process when developing experiential sites to integrate students to become competent, practice-ready pharmacists. As new rotation sites are added or new preceptors are onboarded to oversee pharmacy students, it is important for experiential programs to have tools to educate these additions on how to integrate learners. The methodology of using a 60% threshold can be considered a limitation of this work, as the cutoff is a subjective number based on the variety of inpatient experiential settings used within the participating programs. Experiential settings for both rotation types included large academic teaching hospitals, community hospitals, federal hospitals, state hospitals, for-profit hospitals, non-profit hospitals, and rehabilitation hospitals. For HS only, long-term-care pharmacies were also used.

Of note, with postgraduate year one (PGY-1) residency training in the U.S., the PGY-1 resident must be evaluated on each residency goal (competency) at least once within the residency year, and the resident must be documented as achieving 80% of all goals by the end of the year. We applied a similar approach but adjusted to a >60% APPE competency completion threshold, as each APPE is, at minimum, 4 weeks in length and designed to get students ready to become an entry-level pharmacist. The participating programs aim to have all students develop in the four competency areas of Hospital/Health Pharmacy Systems, Medication Safety and Quality, Clinical Applications, and Professional Practice while on inpatient APPEs, but the programs do not require that every sub-competency is achieved. For residency training, the structure is more robust in competency requirements and generally accepted as the equivalent of 3 to 5 years of work experience in the U.S. Having that higher threshold in residency training is viewed as appropriate by the participating programs.

It is important to mention that the 2015–16 AACP Academic Affairs Standing Committee was charged with identifying and establishing Entrustable Professional Activities (EPAs) for all new U.S. PharmD graduates, and these EPAs were published for pharmacy educators in 2017 [11]. Since then, EPAs for pharmacy graduates have been further revised by AACP members and integrated into the *draft* 2025 ACPE Standards as requirements for PharmD educational outcomes [2,12]. Outlined EPAs by the AACP are essential tasks that trainees can be entrusted to perform independently once trainees have attained sufficient competence [11]. EPAs are divided into six different domains, and there are examples of supporting tasks for each EPA. EPA domains do not demarcate setting-based competencies; therefore, they do not outline rotation-specific competency attainment within the four core required rotation types. Based on current draft standards, the onus is still on the school to delineate rotation-specific competencies.

Findings show that 19 of the 26 sub-competencies are statistically significant in being achieved on one inpatient APPE over the other. When blending rotations, programs can use the statistically significant results to demarcate which sub-competency is contributing to a specific practice area. For example, learner time spent related to demonstrating aseptic techniques should be demarcated to supporting HS practice area activities.

There are strengths and limitations within this study. Strengths include surveying a large cohort of students (n = 168) from two C/SOPs, and data were used only from students who had completed a single inpatient core APPE at the point of data collection. Using data from a single inpatient rotation model, standalone APPE HS and APPE GM rotations, was essential to being able to delineate rotation-specific competencies and sub-competencies. This information can then be used to decipher which activities support rotation-specific outcomes when using blended and longitudinal inpatient APPE models. Additionally, each preceptor was surveyed to attest to trainees’ reporting about completing a specific competency/sub-competency while on their prior core inpatient APPE. Students were asked to self-report, which can introduce potential bias, and the preceptor attestation of student responses was an effort to avert bias. Preceptors on the second inpatient APPE rotation were aware of the student reporting and were advised to provide competency-specific tasks and projects if they felt there were inaccuracies in competency development reporting. Another identified limitation is that the data collected are not capturing whether a sub-competency was or was not offered at the site; they solely capture whether students did or did not achieve the competency. This is an opportunity for future research.

It is important to note the rationale for student participation in this study. The data were collected in March 2020 when Massachusetts U.S.A. became a global hotspot for COVID-19. Major health systems in the area needed to preserve personal protective equipment for hospital and health system employees, and they inevitably could no longer accept onsite experiential learners. Students who were asked to complete this survey were learners who needed only one more rotation (240 h) to graduate from pharmacy school. The experiential office used COVID-19 guideline flexibility allowed by U.S. PharmD accreditors to support students in meeting competencies and addressing any gaps they may need to meet to graduate. The students’ last rotation (March–April 2020) was shifted to remote learning as allowed by accreditors, but the programs needed to ensure compliance with students gaining essential competencies in their experiential learning. The reporting by students was collected to identify what competencies they had already obtained onsite in their prior inpatient rotation, and the remote rotation was developed to have competency-specific projects and activities included for any gaps identified. Data collected were intended for accreditation purposes and quality improvement. The authors submitted a retrospective IRB to use the onsite data captured in this quality assurance process to later support blended and longitudinal inpatient competency delineation, knowing there was an identified gap in the literature.

Based on *draft* 2025 ACPE Standards, the same four core APPEs will still be required for all U.S pharmacy students during their last year of education [2]. As of now, the draft standards do not provide distinct requirements for inpatient and outpatient core APPEs and do not provide an experiential-specific guidance document. C/SOPs can use published competency information from the ASHP-ACPE *Entry-level Competencies Needed for Pharmacy Practice in Hospitals and Health-Systems* Task Force, from the AACP EES work on essential elements, and from the AACP Academic Affairs Committee work on EPAs, to help guide the delineation of core experiential components while offering blended or longitudinal APPE models using a similar approach to this study.

## 5. Conclusions

Data from this study can assist C/SOPs to help delineate rotation-specific APPE inpatient outcomes when using blended or longitudinal APPE models. Data can also support experiential departments in preceptor and site development as a guide to identify sub-competencies that are likely to be achieved in particular inpatient experiences. C/SOPs using these findings could implement core competency delineation using sub-competencies that are significant (*p* < 0.05) when using blended models or longitudinal models. In standalone rotation models, having a competency threshold set is useful in the quality assurance of APPEs; the participating programs utilize >60% as a threshold.

EPA-based evaluation does not account for blended models or provide a delineation of core rotation competencies by setting [11,12]. EPAs are being adopted as PharmD educational outcomes within U.S. pharmacy education, as outlined in *draft* 2025 ACPE Standards [2]. Further research using EPA-supporting tasks published by the AACP to delineate, in a comparable manner to this study, core rotation-specific activities is a research opportunity to expand upon. These data allow for defined emphasis on specific inpatient rotation competencies during continuous quality improvement processes for blended and longitudinal APPE models.

## Figures and Tables

**Table 1:** Hospital/Health Pharmacy Systems.

Sub-Competency *	HS		GM		*p*-Value	Above 60%
	%	n	%	n		
Demonstrate aseptic technique and describe processes and facilities needed to provide sterile compounded parenteral solutions, including the basic requirements of United States Pharmacopeia (USP) 797.	77%	66	35%	29	0.001	HS
Describe the appropriate use of injectable medications, including intravenous, intrathecal, intraocular, intradermal, and other routes. Description should include unique preparation techniques, concentration considerations, rates of administration, special infusion devices, and compatibility considerations.	74%	64	54%	44	0.006	HS
In real or simulated scenarios, supervise pharmacy technicians in their work in medication preparation and delivery.	76%	65	45%	37	0.001	HS
Describe the medication use process in health systems, including how pharmacy impacts the safety of storage, prescribing, transcription, dispensing, administration and monitoring steps.	95%	82	82%	67	0.007	Both
Describe the basic drug procurement process including drug selection, inventory, management, back orders, recalls, drug waste, handling of drug shortages, and their relationship to safe, effective patient care.	91%	78	61%	50	0.001	Both
Describe the integration and interface of clinical and distributive functions, including the synergy that translates into safe and effective medication therapy.	77%	66	74%	61	0.858	Both
Outline the basic functionality of commonly used automated systems related to medication use (such as automated dispensing cabinets, computerized prescriber order entry systems, bar code medication administration systems, programmable infusion devices, and robotics), understanding their appropriate and safe use as well as unintended consequences.	90%	77	65%	53	0.001	Both
Perform activities within a typical hospital drug distribution system, including order receipt, evaluation and review, and describe the appropriate roles of pharmacy technicians and pharmacists in these processes.	90%	77	68%	56	0.001	Both

* Sub-competencies listed in this table are from Appendix C of the 2016 ACPE Standards Guidance Document [10].

**Table 2:** Medication Safety and Quality.

Sub-Competency *	HS		GM		*p*-Value	Above 60%
	%	n	%	n		
Summarize current National Patient Safety Goals and articulate those goals that relate to medication use, pharmaceutical care, and pharmacy’s role in each.	83%	71	60%	49	0.0011	HS
Employ performance improvement techniques used in health systems and describe how they are used to improve the medication use process	76%	65	54%	44	0.0001	HS
Describe how organizations, such as the Joint Commission strive to ensure quality of healthcare through the accreditation process, giving examples of relevant standards related to safe and appropriate medication use.	77%	66	63%	52	0.0589	Both
Describe those national standards, guidelines, best practices, and established principles and process related to quality and safe medication use (e.g., storage of look-alike/sound-alike medications, high-alert medications, storage of concentrated potassium in patient-care areas, dangerous abbreviations, leading decimal points and trailing zeros, quality measure related to medications, etc.).	85%	73	61%	50	0.0005	Both
Given a real or simulated case of a patient transitioning from one care setting to another, effectively reconcile his/her medications and make appropriate communications to involved pharmacy providers.	74%	64	84%	69	0.1205	Both

* Sub-competencies listed in this table are from Appendix C of the 2016 ACPE Standards Guidance Document [10].

**Table 3:** Clinical Applications.

Sub-Competency *	HS		GM		*p*-Value	Above 60%
	%	n	%	n		
Make useful contributions to the establishment of medication-use policies, criteria, and maintenance of the formulary as a student member of the Pharmacy and Therapeutics Committee using an evidence-based approach to evaluation of the literature.	74%	64	54%	44	0.0050	HS
Given a drug information question, access appropriate drug information resources, including primary literature, and provide an accurate and credible answer. Present the answer successfully in both written and oral forms.	79%	68	96%	79	0.0002	Both
Given a real or simulated case requiring practical application of pharmacokinetic dosing principles for commonly used drugs that rely on serum levels for dosing, determine the correct dose.	77%	66	90%	74	0.0189	Both

* Sub-competencies listed in this table are from Appendix C of the 2016 ACPE Standards Guidance Document [10].

**Table 4:** Professional Practice.

Sub-Competency *	HS		GM		*p*-Value	Above 60%
	%	n	%	n		
Demonstrate effective verbal and written communications to staff, patients, and healthcare team members.	90%	77	95%	78	0.1755	Both
Demonstrate professional behavior (attitude, dress, appearance, etc.) in practice settings.	93%	80	94%	77	0.8179	Both
Given a real or simulated case, document appropriate therapeutic recommendations related to medication therapy.	79%	68	95%	78	0.0021	Both
Accurately triage multiple patient-care priorities in times of high activity and workload.	67%	58	77%	63	0.1754	Both
Given a real or simulated case, respond to questions with the appropriate level of detail necessary to ensure proper patient care and communication with other relevant parties.	77%	66	93%	76	0.0043	Both
Given a real or simulated pharmacy-related problem, demonstrate effective problem-solving skills.	81%	70	93%	76	0.0302	Both
Given a real or simulated case, demonstrate an appropriate level of clinical knowledge related to medications and therapeutics in making decisions or recommendations.	77%	66	95%	78	0.0007	Both
Analyze a recently published study.	71%	61	94%	77	0.0001	Both
Describe the impact of pharmacist involvement on medication safety and quality using appropriate literature.	74%	64	78%	64	0.5808	Both
Evaluate medication-use patterns in a specified patient population.	71%	61	67%	55	0.5888	Both

* Sub-competencies listed in this table are from Appendix C of the 2016 ACPE Standards Guidance Document [10].

## Data Availability

The data presented in this study are available upon request from the corresponding author. The data are not publicly available due to academic privacy.
